# ‘I Do Not Want to Depend Too Much on My Children’: Motivations and Challenges for Intergenerational Co-Residence Among Older Adults in Indonesia

**DOI:** 10.1007/s10823-026-09602-y

**Published:** 2026-10-01

**Authors:** Lathifah Hanum, Peter Newcombe, Fivi Nurwianti, Theresa Scott

**Affiliations:** 1 University of Queensland, Brisbane, Australia; 2 Universitas Indonesia, Depok, Indonesia

**Keywords:** Indonesia, Intergenerational co-residence, Older adults, Preferences, Vignette

## Abstract

In Indonesia, intergenerational co-residence in later life is widely regarded as a normative arrangement, yet older adults’ own motivations for and reservations about living with their adult children remain underexamined. This study explored how older Indonesian adults perceive co-residence with their adult children and what considerations shape their preferences. Using a vignette-based qualitative design, we conducted semi-structured interviews with 20 older adults (aged 60 to 78) who were themselves living in multigenerational households. Data were analysed using a hybrid inductive-deductive thematic analysis. When responding to the vignette, most participants (13 of 20) expressed a preference against prioritising co-residence, despite all currently living with an adult child. A recurring motivation was their wish not to become a burden on their children and to preserve their independence. Five factors shaped these perspectives: financial circumstances, relationship quality, filial responsibility, health-related housing suitability, and marital status. By showing that older adults embedded in a strongly pro-co-residence culture nonetheless voice reservations about it and prioritise not becoming a burden on their children, this study challenges the assumption that normative co-residence reflects older adults’ own preferences and suggests that concerns about autonomy and dependency shape later-life living arrangements beyond individualistic Western settings.

## Introduction

Intergenerational co-residence in later life can be an important mechanism for providing care and support to the older generation. As health declines with age, older adults often rely on their adult children for assistance with daily routines, and numerous studies have identified health factors as one of the primary reasons older adults choose to live with their children (Isengard & Szydlik, 2012; Johar et al., 2015; Korinek et al., 2011; Kumar & Williams, 2021; Seltzer & Friedman, 2014; Zhang et al., 2014). In these arrangements, children may take on a more active caregiving role, ranging from supervision to intensive support, depending on the severity of illness.

However, co-residence can also reflect a downward flow of support from parents to adult children. Some adult children return to the parental home not because their parents need care, but because of their own economic or housing difficulties, becoming “boomerang children” (Casares & White, 2018; Takagi et al., 2007). While many parents are accommodating, some begin to feel strained when this support extends over a long period (Tosi & Grundy, 2018). Indeed, in many Western countries, co-residence no longer primarily reflects the frailty of the older person. Research in the United States shows that older adults have become more financially independent of their adult children, while younger adults have become more financially dependent on their parents, and that intergenerationally extended households tend to benefit the younger generation more than the older one (Kahn et al., 2013). Such arrangements may also affect parents’ own well-being. Parents aged 50 and older show increased depressive symptoms after an adult child moves in, particularly when that child is out of work (Caputo, 2019), and older mothers, who often remain the household’s primary caregivers, may be especially affected (Schwarts & Ayalon, 2015).

In many Western contexts, co-residence is less normative and tends to arise in response to particular needs rather than as a default expectation. Comparative European evidence indicates that older parents and adult children are more likely to share a household under conditions of economic insecurity, while living separately remains the more common arrangement (Isengard & Szydlik, 2012). Older adults in these settings often prefer “intimacy at a distance”, maintaining close ties and frequent interaction with their children while retaining their own residential independence (Rosenmayr, 1968).

The Indonesian context differs markedly. Co-residence between older adults and their children is common and largely normative: around 12% of Indonesia’s population are older adults, and approximately 70% of them live in multigenerational households with children, in-laws, and grandchildren (Badan Pusat Statistik 2024b). Because adult children in Indonesia often never leave the parental home, such arrangements would not be characterised as a “boomerang” phenomenon. Instead, children are often expected to remain in the parental home and care for ageing parents, with caregiving frequently, though not exclusively, expected of daughters (Schröder-Butterfill & Fithry, 2014; Surachman et al., 2018). This expectation is echoed across Southeast Asia (Domingo & Asis, 1995; Teerawichitchainan et al., 2015). Yet a normative pattern does not imply uniform preferences: older adults’ own motivations for, and reservations about, co-residence remain varied and underexamined.

These varied preferences can be understood through Intergenerational Solidarity Theory, which frames family cohesion across associational, affectual, consensual, functional, normative, and structural dimensions (Bengtson & Roberts, 1991), and through Social Exchange Theory, which holds that individuals weigh the benefits and costs of their relationships (Homans, 1958). Together, these lenses suggest that co-residence may be a deliberate, reciprocal strategy rather than a simple response to need. Despite the prevalence of co-residence in Indonesia, however, older adults’ own perspectives on it have received limited attention. The present qualitative study uses vignette-based interviews to examine how older adults reason about living with their adult children, focusing on the personal and interpersonal considerations that shape these decisions, and thereby offers insight into intergenerational support that can inform cross-cultural understandings of ageing.

## Methods

### Study Design

A vignette-based qualitative approach, combined with semi-structured interviews, was used to explore older adults’ perspectives on intergenerational co-residence. The vignette illustrations were developed through focus group discussions with Indonesian adults (60+) living with their adult children. Data were analysed using a hybrid thematic analysis combining deductive and inductive coding (Fereday & Muir-Cochrane, 2006). This hybrid approach has been applied across qualitative research in a range of fields, such as Zhou et al. (2023) and Scott et al. (2023).

### Participants and Procedure

The researcher recruited 20 older adults (aged 60+) engaged in intergenerational co-residence using convenience sampling. Recruitment advertisements were shared via social media (Facebook, Instagram) and older adult communities, including Older Adults School (*Sekolah Lansia*), a program by Indonesia’s National Family Planning Coordinating Board to support the development needs of older adults, allowing them to grow together with others in their age group (Badan Kependudukan dan Keluarga Berencana Nasional (BKKBN), 2024). Individuals who expressed interest completed a written informed consent form via Qualtrics, administered by the first author, before being contacted to schedule the interview. Efforts were made to include participants from diverse locations across Indonesia to capture varied perspectives. Participants’ kinship systems were identified from their own accounts during the interviews rather than ascribed based on their region of residence, and the resulting sample comprised bilateral, patrilineal, and matrilineal backgrounds. Given the small sample size, this diversity was intended to capture a range of cultural perspectives rather than to support systematic comparisons between kinship groups. Potential participants completed a brief survey collecting demographic data and health status, assessed using the ADL and IADL scales adapted for Indonesia (Kementerian Kesehatan, 2016). Eligibility required an ADL score of at least 12 (no more than mild dependency) and an IADL score of at least 2 (occasional assistance). This lower threshold was applied so that participants’ health was not so poor that health concerns would overshadow other considerations, allowing the exploratory analysis to surface the broader range of factors shaping co-residence decisions. No upper limit on functional ability was imposed; in this sample, ADL scores ranged from 18 to 20 and IADL scores from 6 to 8, indicating that participants were largely independent in their daily functioning. Interviews, lasting 45–60 min, were conducted in Indonesian and audio-recorded. Of the 20 interviews, 17 were conducted face-to-face (7 by the first author, a clinical psychologist experienced in working with older adults in Indonesia, and 10 by five trained field research assistants in Bali, Pontianak, Samarinda, Makassar, and Manado), and 3 were conducted online via Zoom by the first author. Data were collected between September 2024 and February 2025. Table 1 presents participant details.

**Table 1:** Characteristics of participants

Pseudonym	Age	Sex	Lives with	Region (island)	Kinship system	SES
Teguh	74	M	Spouse, children, grandchildren	Java	Bilateral	High
Maya	60	F	Spouse and children	Java	Bilateral	Middle
Carla	71	F	Children	Java	Bilateral	Middle
Tuti	63	F	Spouse and children	Java	Patrilineal	Middle
Sarah	72	F	Spouse and children	Java	Bilateral	High
Adi	75	M	Spouse, children, grandchildren	Java	Bilateral	Low
Nina	60	F	Spouse and children	Sumatra	Patrilineal	Low
Rara	65	F	Spouse and children	Java	Bilateral	High
Puspita	61	F	Spouse and children	Java	Bilateral	Middle
Binda	67	F	Spouse, children, grandchildren	Sumatra	Matrilineal	Middle
Shinta	78	F	Children	Kalimantan	Bilateral	Low
Asri	63	F	Spouse and children	Kalimantan	Bilateral	Low
Tina	61	F	Children	Sulawesi	Bilateral	High
Lily	64	F	Spouse and children	Sulawesi	Bilateral	High
Eko	65	M	Spouse, children, grandchildren	Bali	Patrilineal	Low
Ani	69	F	Children	Bali	Patrilineal	Low
Tom	71	M	Spouse and children	Sulawesi	Bilateral	Middle
Edo	68	M	Spouse and children	Sulawesi	Bilateral	Low
Andy	61	M	Spouse and children	Kalimantan	Bilateral	Low
Gandy	68	M	Spouse and children	Kalimantan	Bilateral	Low

Kinship system reflects the system participants themselves identified with during the interviews, rather than one ascribed based on their region of residence. SES categories are based on average monthly household expenditure (in thousand IDR): Low (< 500), Middle (500-1,499), and High (≥ 1,500)

Interviews began with a vignette about Mr Mamat, a man in his 70 s who lives with his wife in their home, while his adult children live independently elsewhere. Despite being in good health, he worries about the future and wishes for one child to return home. Participants were asked whether one of his adult children should choose co-residence and to justify their reasoning, and were then invited to reflect on their own circumstances. The vignette depicted a scenario in which the older adult was not currently co-residing with an adult child, a situation different from participants’ own arrangements. This distance from their lived experience was intended to invite reasoning about the decision itself, rather than prompting justification of an arrangement already in place. Because co-residence is normatively expected in Indonesia, discussing a hypothetical family allowed participants to express reservations that might be difficult to voice about their own children without appearing critical of them. Following the vignette, the semi-structured interview focused on participants’ motivations for living, or not living, with their adult children, with follow-up probes used to explore the reasons behind their responses.

### Data Analysis

The analysis employed a hybrid thematic analysis following Fereday and Muir-Cochrane (2006), combining deductive and inductive coding. This hybrid approach integrates the data-driven inductive coding approach of Boyatzis (1998) with the deductive, a priori template-of-codes approach of Crabtree and Miller (1999). Deductive codes were derived from factors identified in prior research (Hanum et al., 2024), namely financial circumstances, health conditions, kinship systems, marital status, education, and family size. In contrast, inductive codes were developed from participants’ accounts to capture influences not anticipated by that framework. A code manual was developed from an initial subset of interviews and tested by having two transcripts coded independently; as no substantive discrepancies emerged, the first author then coded the remaining data systematically. Interviews, conducted in Indonesian, were transcribed verbatim by the first author, which supported close familiarity with the data, and transcripts were also reviewed by the third author. The first author recorded memos throughout the analysis. Each transcript was examined individually, identifying commonalities while attending to participants’ subjective experiences, and coding was iterative, with codes refined until consistent patterns emerged from which the themes were derived. Two professional translators provided English translations of the quotations, and the analysis was supervised by the second and last authors. Regular meetings among the authors supported discussion of the findings, with records maintained to ensure transparency.

## Results

This study analysed data from 20 participants aged 60 to 78 years (M = 66.8, SD = 5.39), all residing in multigenerational households. Thirteen of the 20 participants were women, and most lived with their spouse and an adult child, while four lived solely with their adult children and another four in three-generation families. Nearly all of the participants owned their homes. Among those co-residing with an adult child, some lived with children who had migrated for education and later returned home after completing their studies; these children were now employed and financially independent, though unmarried. Participants did not report specific plans for whether these children would remain in the family home following marriage, though most also had other, already-married children living separately. This suggests that some co-residential arrangements observed here may still be subject to change, even where they currently appear settled. More broadly, as the following section shows, several participants’ current arrangements had arisen from circumstances rather than reflecting their own preferred arrangements, a distinction that becomes especially relevant when interpreting participants’ stated preferences later in this section.

When reflecting on the vignette, most participants expressed a preference against prioritising co-residence with their adult children: 13 indicated they would not choose to live together, 6 favoured co-residence, and 1 remained undecided. This is notable because all participants were themselves currently living with an adult child. A recurring thread across these accounts, developed below, was participants’ wish not to become a burden on their children. This preference was not confined to the most economically secure participants. It was expressed by 6 of the 9 participants in the lowest expenditure category, 5 of 6 in the middle category, and 2 of 5 in the highest expenditure category, indicating that a reluctance to prioritise co-residence was voiced across socio-economic positions rather than only by those best able to live independently.

Thematic analysis identified five factors shaping these perspectives: financial circumstances (20 of 20 participants), relationship quality and filial responsibility (each 19 of 20), health-related housing suitability (18 of 20), and marital status (11 of 20). Table 2 presents these themes with their definitions, constituent codes, and the number of participants expressing each.

**Table 2:** Themes and their constituent codes, with participant frequencies

Theme	Definition	Codes	Participants (*n* = 20)
Filial responsibility	Parents’ perceptions of their adult children’s roles and obligations in parental care, shaped by cultural and social values and by how many family members share caregiving.	Kinship system; number of family members; presence of grandchildren	19
Relationship quality	The emotional closeness, communication, and mutual understanding between older adults and their children, children-in-law, and grandchildren that support harmony in shared living.	Open communication; emotional support and trust; involvement in family decisions; companionship beyond the immediate family	19
Marital status	The marital status of older adults and of their adult children, and how it shapes their preference for intergenerational co-residence.	Own marital status (married, widowed, divorced); children’s marital status; spousal involvement in the decision	11
Financial circumstances	Older adults’ economic situation, including income, savings, and assets, and how it shapes their capacity to live independently or to support their adult children.	Financial independence; sharing living costs; financial support to children; children’s employment	20
Health-related housing suitability	How older adults evaluate their living environment in light of their health, including access to healthcare and their familiarity of their surroundings.	Health conditions and mobility; proximity to health services; suitability and familiarity of the living environment.	18

Frequencies indicate the number of participants (of 20) whose accounts contributed to each theme, not the number of coded excerpts. Illustrative quotations are presented in the Results

### Filial Responsibility

Filial responsibility was the most widely shared concern alongside relationship quality, expressed by 19 of the 20 participants. It centred on which family members were expected to provide care, and on how cultural and religious values shaped those expectations. Several participants noted that a married daughter’s capacity to care for her parents depended on her husband’s consent, reflecting the household authority of men in many Indonesian families and the financial demands that caregiving entails.

Nina: While a daughter may be willing to care for her parents, it ultimately depends on her husband’s approval. Therefore, it’s a sensitive issue that requires careful consideration.

Participants’ own histories shaped what they expected of their children. Those who had grown up in three-generation households sometimes wished for similar care in later life, whereas those who had themselves cared for a frail parent were often reluctant to see their children repeat that experience. For the latter group, smaller gestures of attention were considered sufficient, and their reluctance was expressed less as a lack of entitlement than as a deliberate choice not to impose.

Tuti: Yes, yes, yes. I took care of my mother at home, but it was not to pressure my children to do the same thing. […] Although we are financially sufficient if they transfer us money monthly to pay for the electricity bills, it is a form of attention. So, we think that we are being cared for. I am not talking about the amount of money, but I am talking about the children’s attention.

Participants were also aware that their children face demanding careers, and they framed their own restraint in these terms, treating their children’s economic prospects as a reason to limit what they asked of them.

Andy: Indeed, the child’s career and economic well-being are crucial. Living with parents might put their job and financial prospects at risk. […] We would never stand in the way of our children’s quest for prosperity.

Expectations were rarely directed at one child alone. Participants differentiated between sons and daughters in line with religious or kinship norms, with Muslim participants noting that sons bear primary responsibility, and a Minangkabau participant describing daughters as carrying most caregiving duties under a matrilineal system. In practice, however, participants qualified these norms rather than applying them strictly, treating caregiving as a duty shared across the family.

Binda: As for a Minang person, they tend to do everything with their daughters. The family follows the mother’s lineage. However, there are still things daughters cannot fulfil, and they have to be assisted by the sons. Sons-in-law need to be involved with these kinds of businesses, too.

A minority held to a more traditional view. One male participant who had grown up in a multigenerational household regarded three generations under one roof as the proper form of family life and wished his child to remain at home after marriage, illustrating that the preference against co-residence was not universal in this sample.

Adi: It depends on the person and the family. For me, ever since I was young, my grandfather and grandmother have lived in our house. So, we see that a proper family is like that.

Taken together, these accounts suggest that filial responsibility in this sample was not tied to rigid, unidirectional expectations. Gender roles in caregiving were treated as flexible, with daughters encouraged to pursue careers and sons free to seek work elsewhere, and responsibility was distributed across the family rather than assigned to a single child.

### Relationship Quality Between Older Adults and Family Members

Alongside cultural and religious norms, participants weighed the quality of their family relationships, a consideration raised by 19 of the 20 participants. Communication was central: participants treated the capacity to speak openly with their children and children-in-law as the foundation of trust and as a precondition for living together well. Because co-residence multiplies daily contact, they regarded mutual understanding, particularly with in-laws, as essential to household harmony.

Trust in their children, however, did not automatically translate into confidence about co-residence. Several participants who were already living with an adult child said they would be reluctant to move into a child’s home elsewhere, since doing so would sever ties with the extended family and neighbours they relied on when their children were occupied with work. In responding to the vignette, one participant read Mr Mamat’s situation through his own surroundings.

Tom: Parents here love when their children come to visit and live with them. However, if they are told to move out to the child’s house, I do not think so. They already feel at home here. […] Most members of the extended family live here, so everything is easy. […] Mr Mamat most likely experiences that, too. 

Participants also valued being able to express their views freely, while acknowledging that two-way communication is not always easy when parents and children reason from different experiences. Notably, several described guiding their children’s decisions rather than treating the conversation as an exchange between equals.

Eko: Communication is key. My son and I have regular conversations. […] I’m always careful to use language that’s accessible to them despite my greater experience. As a teacher who frequently attends trainings, I aim to be a role model and share wisdom on parenting from my readings. […] This guidance is especially pertinent for my daughter-in-law, given that she came from a different family culture.

Expressing disagreement constructively mattered as much as agreement, since closer contact between parents, children, and in-laws can generate friction. Most participants, both men and women, maintained a distinction between children and children-in-law, reasoning that a daughter-in-law’s position and feelings needed separate considerations; for instance, several noted that if Mr Mamat’s son were to co-reside with him, his wife’s relationship with her daughter-in-law would also need to be taken into account. Some participants addressed this by explicitly rejecting the conventional distinction between children and children-in-law, which allowed them to address problems with both without appearing to take sides.

Binda: In my house, my son-in-law seems like my son already. […] If they make a mistake, I will let both the husband and wife know. […] I think that is an old paradigm that considers our in-laws as outsiders. In-laws are our children. Once they marry one of our children, they become ours too.

Participants further emphasised being included in family consultations, engaging their children in discussions before major decisions, and seeking to balance their own care needs with their children’s plans.

Lily: We’ve always taught our children to be forward-thinking, especially about how they’ll care for us as we age. […] They’ve given it considerable thought and have a plan in place, ensuring one of them is always close enough to come home if needed. It’s not about obligation; it’s about their willingness to consider our future needs.

Finally, participants framed emotional support as something they gave as much as received. Remaining self-sufficient was itself described as a form of support, freeing their children to concentrate on their own lives, which suggests that within these households the exchange of support was understood as running in both directions rather than flowing only towards the older generation.

### Marital Status

Marital status was raised by 11 of the 20 participants, making it the least widely shared of the five themes, and it concerned the marital status of the participants themselves as well as that of their adult children. Participants with a living spouse generally felt less need to live with a child, while the marital status of the child introduced a further consideration, since co-residence with a married child also meant living with a son-in-law or daughter-in-law.

This consideration was strongly gendered. Mothers were reluctant to live with married sons because they anticipated friction with a daughter-in-law, whereas fathers rarely raised comparable concerns about sons-in-law. Participants attributed this asymmetry to the division of domestic work: mother-in-law and daughter-in-law are both responsible for running the household and must therefore reconcile different ways of doing so, while fathers-in-law and sons-in-law share no such overlapping domain.

Adi: In a family, there is a power struggle. The power struggle usually happens between a mother and her daughter-in-law. One of them should give in. If they want to live under the same roof, then one of them should give in.

Widowhood shifted this calculation. Participants who anticipated being left alone regarded co-residence more favourably, describing it as protection against loneliness and as a practical arrangement for monitoring a parent’s well-being. Some participants noted explicitly that the protagonist’s situation would change if his wife were no longer alive.

Tuti: Mr Mamat’s wife is still alive. However, if one of them passes away, it is more likely for the remaining parent to live with their child. […] So, to take care of the parents and be more alert, the best option is to take them to live with the child.

Among the six participants who favoured co-residence in the event of widowhood, preferences about who would relocate were mixed: four expected a child to return to the family home, while the remaining two, echoing Tuti’s reasoning for Mr Mamat, indicated a willingness to relocate to a child’s home themselves so as to minimise disruption to their child’s work and routine.

These accounts indicate that the preference against co-residence expressed by most participants was conditional rather than absolute. It rested in part on having a spouse and on being able to avoid the domestic tensions that co-residence with a married child can produce, and participants recognised that the balance could shift with widowhood or declining health. Among participants whose co-resident child was already married, several attributed the current arrangement to circumstances specific to the child rather than to their own preference: one recounted that the child had previously lived separately but returned when the parents’ home proved more convenient for work, while others described their child needing support, whether with childcare for grandchildren or during a period of financial difficulty. These accounts suggest that many participants’ current co-residence reflects circumstances that had already arisen, often centred on the child’s needs, rather than representing their own preferred arrangement.

### Financial Circumstances

Financial circumstances were raised by all 20 participants, making this the most widely shared consideration. Participants weighed their retirement savings, assets, and above all homeownership, and those with sufficient space or resources were generally more open to co-residence, since they could accommodate a child without either household losing its autonomy. A larger home allowed married children to run their own affairs and reduced the daily friction, particularly between mothers-in-law and daughters-in-law, that arises when kitchens and common spaces must be shared.

Financial security was described first as the basis of independence. Participants emphasised careful planning, saving, and preserving assets so that they could sustain themselves without drawing on their children, and several were explicit that living apart was a way of protecting that autonomy rather than a sign of weak family ties. Homeownership also worked in the opposite direction, as a resource participants could extend to children who struggled to afford housing of their own. Where their means were limited, participants expected some reciprocity, such as help with household costs, and in responding to the vignette one participant framed this as an ordinary exchange rather than dependence.

Adi: Usually, grandparents’ income has been significantly reduced. Maybe it is reduced, or they do not have income at all. They are retirees, right? Mr Mamat can obtain financial support from his children. Let us assume that the children are paying rent in their parents’ house.

Assets also conferred a degree of influence. Participants with substantial property retained some say over their children’s living arrangements even when those children were financially independent and married, and several intended to leave the family home to whichever child was willing to co-reside, treating the house as both a recognition of that child’s support and an inducement to provide it.

Asri: I’ve always encouraged my children to lead independent lives. Currently, I am in good health. However, should my husband and I become ill, we’ve asked our children to be prepared to return home to care for us. This has also been discussed with their spouses, addressing the question of who might wish to return and inherit the family home in the future.

Participants with fewer assets reasoned differently. Unable to offer material support, they encouraged their children to build careers and financial stability first and framed their own patience as a contribution to their children’s prospects rather than a resignation.

Ani: If I look back at myself, when my children are living out of town, there is a possibility that they are committed to being successful first before returning home and taking care of me. […] If we live together and we have nothing, what can we eat at home? […] If they return one day, I will be grateful, only if they can. The hope and will are there, but it is not the right time yet.

Financial standing thus shaped co-residence in two directions at once. It enabled participants to remain independent and to avoid burdening their children, while also giving those with assets a measure of leverage over where and with whom their children lived, so that financial security underpinned both their restraint and their continuing authority within the family.

### Health-Related Housing Suitability

Health-related housing suitability was raised by 18 of the 20 participants. Their reasoning extended beyond whether their children could care for them to the condition of their home and, just as importantly, the environment around it. This consideration was particularly salient given the vignette’s detail, with Mr Mamat’s declining health requiring regular medical check-ups; participants reasoned that relocating would disrupt his familiarity with established healthcare routes and providers, and many extended this reasoning to their own circumstances. Proximity to hospitals, health centres, and older adult communities supported both physical and mental well-being, and participants who were well served in these respects were markedly reluctant to relocate to a child’s home, where they would have to rebuild routines and relationships from scratch.

Participants recognised that their health could decline and that a sudden deterioration might require intensive assistance, which for some was a reason to want a child nearby (see quote by Eko, below). Yet the same anticipation produced the opposite conclusion for others, who worried that their care needs would fall on children already stretched by work, and who therefore treated maintaining their own health and self-sufficiency as a way of protecting their children.

Shinta: Living alone isn’t an issue. However, we must consider that our children have their own work commitments. […] In the past, staying close to our parents was customary, but times have changed. Now, if we’re able and healthy, striving for self-reliance is preferable. […] Nearing 78, I see my child working hard, often late into the evening. Meanwhile, we’re still robust and cherish our independence.

Where participants did want a child close at hand, they framed it as a mutual arrangement rather than a claim. Several said they would release a child to work elsewhere if opportunity required it, and would consider moving closer to their children themselves, treating proximity as something to be negotiated between two sets of needs.

Eko: Life-wise, we’re content with our separate spaces. I am downstairs, and they’re upstairs. My main concern is for the times when I might be ill and need assistance. […] If he were farther away, I’d have to let him go for the sake of his life, much like Mr Mamat did. […] It’s about compromising and seeking the best way for both sides, as my parents did when they moved in with their children.

What made staying put viable was less the house itself than the infrastructure and relationships surrounding it. Participants valued being able to reach medical services on their own rather than depending on a child for transport to routine appointments, and several drew on nearby older adult communities, health centres, and organised activities for exercise and social contact. These local networks substituted for the daily presence of family, and in doing so made independent living sustainable rather than merely preferable.

Ani: We do not have to be with our children all the time. That is why we need to be able to make friends with our surroundings, befriend fellow older people, and share experiences. I go to a school for older people. I also attend exercise at a Public Health Centre near my house. I have a lot of activities with my friends my age.

Continuity of care mattered for the same reason. Moving to a child’s home would mean changing hospitals and doctors and adapting to unfamiliar providers, a prospect participants resisted. Taken together with the loss of neighbours and nearby relatives, this helps explain the study’s central finding: 13 of the 20 participants said they would not choose to live with their adult children, and where co-residence was contemplated, most preferred that a child return to the family home rather than they themselves relocate.

These accounts indicate that what participants sought to preserve was not simply a dwelling but an established ecology of services, routines, and relationships. Familiarity with that setting was what allowed them to manage their own health and to remain, in their own account, independent of their children.

## Discussion

Discussion on intergenerational co-residence often centres on the assumption that older adults primarily seek care from their adult children (Cameron, 2000). This study explored the considerations that shape older Indonesian adults’ perspectives on intergenerational co-residence with their adult children. Although all participants were themselves already living in multigenerational households, most did not regard co-residence as something they prioritised: when reflecting on a vignette concerning hypothetical future co-residence with an adult child, 13 of the 20 indicated they would not choose to pursue that option. Their considerations spanned personal factors, such as financial circumstances and health, and relational factors involving their children and in-laws, as well as more limited observations concerning grandchildren, and were shaped by their cultural backgrounds and life experiences. Yet the thread running most consistently through these accounts was a strong wish not to become a burden on their children, a preference that persisted even though they were currently co-residing.

Five factors influencing older adults’ decisions whether to live in multigenerational households were identified. The first factor was filial responsibility, which encompassed expectations regarding adult children’s role in parental care. Filial responsibility was a central theme, with participants expressing diverse views on how this value was applied within their families. Studies from other Asian countries, such as Taiwan and China, suggest that filial piety traditionally assigns greater caregiving responsibility to sons (Chen et al., 2022; Liu et al., 2012; Yasuda et al., 2011; Zhang et al., 2014). However, participants in this study did not prioritise gender when considering co-residence. For instance, a Minangkabau participant, following a matrilineal kinship system (Halimatussa’diyah et al., 2024), affirmed that although daughters carry most caregiving duties, sons and sons-in-law must also be involved in tasks daughters cannot fulfil. Similarly, participants from North Sumatra or Bali who adhered to a patrilineal system (Absor et al., 2023; Titisari et al., 2023) did not solely place the responsibility for their care on their sons. Even among participants who referenced religious values, such as Islam, which teaches that sons bear primary responsibility for parental care (Wanto, 2017), caregiving was generally viewed as a shared family duty rather than the responsibility of one child. This suggests a shift towards a more collective approach to caregiving, regardless of cultural or religious norms. Comparable work in Bali has shown how local knowledge shapes the way families organise multigenerational caregiving, underlining that these arrangements are worked out within specific cultural settings rather than following a single national pattern (Lestari et al., 2023).

These findings can be further interpreted through the Intergenerational Solidarity Theory, which explains the cohesion of intergenerational family relationships across six dimensions: associational, affectual, consensual, functional, normative, and structural solidarity (Bengtson & Roberts, 1991). In this study, participants demonstrated strong normative solidarity, reflected in their commitment to fulfilling parental roles and maintaining family obligations. At the same time, their willingness to continue supporting their adult children emotionally and practically reflects a high level of functional solidarity, which involves the exchange of support and resources between generations. Rather than expecting care from their children, many participants emphasised the importance of remaining independent and contributing to their children’s growth and well-being. This reflects a reciprocal model of intergenerational support, in which caregiving responsibilities are shared among family members regardless of gender, rather than following a fixed expectation that adult children provide unidirectional care.

Previous research in rural Indonesia found that older adults prioritised living with family members over the quality of their relationships with them (Absor et al., 2023). However, the findings of this study highlight the importance participants placed on relationship quality, particularly when considering co-residence with in-laws. Female participants, in particular, expressed reluctance to co-reside if it risked intergenerational conflict, a concern supported by prior studies on communication challenges between parents and in-laws (Mikucki-Enyart, 2011; Saviet & Greif, 2021). Compatibility with in-laws emerged as a key factor in older adults’ willingness to live with their married children.

Additionally, potential friction between mothers-in-law and daughters-in-law was a consideration for some participants, and many female participants consequently preferred living independently or, if necessary, with their daughters. Research on sons-in-law is limited, but existing studies suggest that parents experience fewer conflicts with them than with daughters-in-law (Globerman, 1996; Greif & Woolley, 2018; Kleban et al., 1989). This likely reflects caregiving expectations in Indonesia, where household responsibilities, including older adults’ care, are traditionally assigned to women in many Southeast and East Asian societies (Ken & Teerawichitchainan, 2015; Zhang & Harper, 2022); daughters-in-law thus bear the brunt of caregiving and friction, whereas sons-in-law, less involved in daily care, report fewer tensions (Kleban et al., 1989).

Many participants expressed a strong desire not to depend too much on their children. To achieve this, some prioritised financial independence from an early age, ensuring they could live without relying on their children’s support, and even hoped to continue aiding their children, including financial help. This aligns with research from China showing that parents are more willing to live with their adult children when financially secure (Meng et al., 2017). Some participants also believed that financial support gave them leverage in their relationships with their children, including the ability to invite them back to live in the family home. Having adequate assets provides older adults with the ability to manage their families, including their adult children (Domingo & Asis, 1995). Previous studies have shown that children are more willing to live with their parents when financial support, such as housing or money, is provided (Han et al., 2020; Smits et al., 2010; VanWey & Cebulko, 2007; Yasuda et al., 2011). Moreover, parents who can offer financial assistance are reported to have better mental health than those who cannot (Lee et al., 2014), and strong financial capabilities also help prevent older adults from feeling like a burden to their children (Gerber et al., 2019; Jennings et al., 2014).

Living together between older adults and their children can facilitate the exchange of support between the two generations. Given the challenges the younger generation faces in owning a home, parents’ willingness to share their living space can be invaluable to their adult children. In such cases, the flow of support runs at least partially downward, from parent to child, so that co-residence serves the child’s needs as much as the parent’s, positioning the older adult as a provider rather than a dependent. At the same time, children can provide more attention to their parents’ needs and care by co-residing (Chai & Jun, 2017; Domingo & Asis, 1995; Keene & Batson, 2010; Kim & Kim, 2003; VanWey & Cebulko, 2007).

Health remained central to how participants weighed these arrangements. Declining health in later life can make co-residence a practical source of daily assistance, yet adult children are simultaneously building careers and families of their own, so the care older adults might need competes with their children’s other commitments. Ethnographic work in urban Indonesia has likewise highlighted the vulnerability that accompanies ill health in later life and the fragility of the care arrangements older people rely on (Van Eeuwijk, 2006). Co-residence therefore appeared less as a straightforward response to need than as a strategic and reciprocal arrangement negotiated between two generations (Cong & Silverstein, 2015; Taniguchi & Kaufman, 2017).

Therefore, older adults in this study made efforts to maintain their independence. They engaged in regular exercise and built strong relationships with peers in their neighbourhoods, and the presence of a community of peers further contributed to their expressed reluctance to move to their children’s homes. Some participants in this study were members of an Older Adults School (*Sekolah Lansia*) and familiar with the activities and resources available around their homes. Familiarity with their home environment helped them feel independent and less of a burden to their children, promoting their psychological well-being (Meng et al., 2017; Waterworth et al., 2019). While they hoped their children would eventually take responsibility for caring for them, this did not mean they wished to become completely dependent or to interfere with their children’s roles and responsibilities. This resonates with evidence from Yogyakarta and West Sumatra that co-residence with an adult child is not always attainable or preferred, and that older Indonesians actively manage the balance between dependence, independence, and interdependence rather than passively occupying a household position (Insriani et al., 2024).

At the same time, participants’ accounts complicate any simple reading of older adults as merely deferential. Alongside their concern not to burden their children, participants voiced clear expectations of them and often spoke from a position of relative authority: they used their financial standing as leverage, including the capacity to invite children back into the family home and positioned themselves as advisors and role models rather than as equal partners in decision-making. Even the value they placed on open communication was framed in these terms, as guidance offered to children rather than a fully reciprocal exchange. The tensions surrounding co-residence with in-laws point in the same direction: in navigating potential conflict, particularly between mothers-in-law and daughters-in-law, older participants asserted their own preferences, for example by favouring co-residence with a daughter or choosing to live independently. Older adults’ willingness to accommodate their children thus coexists with, rather than displaces, a strong sense of their own expectations and standing within the family.

Previous research indicates that older adults in rural areas prioritise co-residence as a form of filial responsibility, with children expected to live with their parents (DaVanzo & Chan, 1994; Gruijters & Ermisch, 2019; Takagi et al., 2007). However, other researchers have shown that urban older adults tend to view filial responsibility as being expressed in various ways, not necessarily through co-residence (Korinek et al., 2011; Ren & Treiman, 2015; Yasuda et al., 2011). This difference may be due to the fast-paced lifestyle in cities, where children and parents spend less time together, whereas in rural areas, where many people work in agriculture, forestry, or fisheries fields (Badan Pusat Statistik, 2024a), parents and adult children tend to interact more frequently. Given that most participants in this study lived in urban areas, their perspectives were shaped by urban family life, where adult children are usually occupied with work demands. In this respect, the urban participants in this study resemble the contemporary Western pattern noted earlier, in which co-residence is no longer driven primarily by the frailty or dependence of the older generation. These participants were largely financially independent and oriented toward maintaining their autonomy, and where co-residence occurred, support often flowed downward to their adult children rather than the reverse (Beard & Kunharibowo, 2001; Kahn et al., 2013; Schröder-Butterfill, 2004).

This study has several limitations. The first is the unequal number of participants from urban and rural areas, which may have influenced how they viewed the enactment of filial responsibility in co-residence. With only four participants from rural areas, the data did not permit a systematic comparison between urban and rural accounts, and the urban-oriented interpretation above should be read with this in mind. Some potential participants from rural areas in Indonesia declined to take part in the in-depth interviews, citing a lack of confidence in their ability to contribute as research participants, and remained hesitant despite personal outreach due to their unfamiliarity with the research process. Future research could benefit from balancing participants from both settings to capture the phenomenon more comprehensively.

A strength of this study was the involvement of five research assistants from different provinces, each collecting data in their own region. Their familiarity with local cultures and community dynamics likely facilitated participant engagement and contributed to a more contextually grounded process. All research assistants had graduated from the Faculty of Psychology with a minimum grade of A in an interview course and received interview training from the first author beforehand. While variations in their prior experience may have influenced their interactions with participants, their regional knowledge and targeted training enhanced the quality and relevance of the data collected.

## Conclusion

Discussion on intergenerational co-residence often centres on the assumption that older adults primarily seek care from their adult children. However, this study identified five key factors that shape older Indonesian adults’ considerations when thinking about life in multigenerational households: filial responsibility, relationship quality with family members, marital status, financial circumstances, and health-related housing suitability. These factors suggest that older adults prioritise maintaining harmonious relationships with their adult children rather than viewing co-residence solely as a means of fulfilling their own needs. In this study, participants seem to actively avoid situations where living with their children might hinder their children’s independence and personal development. More broadly, these findings speak to an international audience: they show that in a setting where co-residence is normative and until recently was near-universal, older adults nonetheless hold reservations about it and actively work to avoid becoming dependent. This cautions against reading high co-residence rates in any culture as evidence of older adults’ preference for it, and suggests that concerns about autonomy and not burdening one’s children shape later-life living arrangements beyond individualistic Western contexts.

This study expands the understanding of intergenerational co-residence beyond traditional caregiving expectations, highlighting how older adults prioritise the quality of their relationships with their families while maintaining their independence and ability to provide support to their children. Rather than being the passive recipient of care, older adults in this study engaged actively in reasoning through the considerations involved in living arrangements, reflecting on their own values and circumstances rather than simply accepting a predetermined caregiving role. This study also offers practical implications for families and communities, encouraging a more mutual approach to living arrangements between older adults and their adult children rather than simply considering the needs of one party.

Additionally, the findings may be useful for stakeholders, including community organisations and social welfare programs, in developing initiatives that align with older adults’ preferences and promote sustainable intergenerational living arrangements. While this study does not directly examine policy development, it provides valuable insights that could inform discussions on ageing and family support systems in Indonesia. This study offers a more nuanced and culturally relevant perspective on ageing and family dynamics by shifting the narrative from obligation-based co-residence to one that acknowledges older adults as active participants in shaping their living arrangements.

## Data Availability

The data supporting the findings of this study consists of anonymized interview transcripts collected from older adult participants in Indonesia. Due to ethical considerations and confidentiality agreements, the data are not publicly available. However, limited access to anonymized excerpts may be granted upon reasonable request and with appropriate ethical approval.
